# Levamisole Suppresses CD4^+^ T-Cell Proliferation and Antigen-Presenting Cell Activation in Aplastic Anemia by Regulating the JAK/STAT and TLR Signaling Pathways

**DOI:** 10.3389/fimmu.2022.907808

**Published:** 2022-07-14

**Authors:** Jiali Wang, Jia Liu, Mingyang Wang, Fei Zhao, Meili Ge, Li Liu, Erlie Jiang, Sizhou Feng, Mingzhe Han, Xiaolei Pei, Yizhou Zheng

**Affiliations:** ^1^ State Key Laboratory of Experimental Hematology, National Clinical Research Center for Blood Diseases, Haihe Laboratory of Cell Ecosystem, Institute of Hematology and Blood Diseases Hospital, Chinese Academy of Medical Sciences and Peking Union Medical College, Tianjin, China; ^2^ Hematopoietic Stem Cell Transplant Center, Institute of Hematology and Blood Diseases Hospital, Chinese Academy of Medical Sciences and Peking Union Medical College, Tianjin, China; ^3^ Anemia Disease Center, Institute of Hematology and Blood Diseases Hospital, Chinese Academy of Medical Sciences and Peking Union Medical College, Tianjin, China

**Keywords:** levamisole, aplastic anemia, immune response, JAK/STAT, TLR

## Abstract

Aplastic anemia (AA) is a life-threatening disease primarily caused by a metabolic disorder and an altered immune response in the bone marrow (BM) microenvironment, where cytotoxic immune cells attack resident cells and lead to hematopoietic failure. We previously reported an efficient strategy by applying cyclosporin (CSA) combined with levamisole (CSA+LMS-based regimen) in the treatment of AA, but the immunoregulatory mechanism of LMS was still unclear. Here, the therapeutic effects of LMS were examined *in vivo* using the BM failure murine model. Meanwhile, the proportion and related function of T cells were measured by flow cytometry *in vivo* and *in vitro*. The involved signaling pathways were screened by RNA-seq and virtual binding analysis, which were further verified by interference experiments using the specific antagonists on the targeting cells by RT-PCR *in vitro*. In this study, the CSA+LMS-based regimen showed a superior immune-suppressive response and higher recession rate than standard CSA therapy in the clinical retrospective study. LMS improved pancytopenia and extended the survival in an immune-mediated BM failure murine model by suppressing effector T cells and promoting regulatory T-cell expansion, which were also confirmed by *in vitro* experiments. By screening of binding targets, we found that JAK1/2 and TLR7 showed the highest docking score as LMS targeting molecules. In terms of the underlying molecular mechanisms, LMS could inhibit JAK/STAT and TLR7 signaling activity and downstream involved molecules. In summary, LMS treatment could inhibit T-cell activation and downregulate related molecules by the JAK/STAT and TLR signaling pathways, supporting the valuable clinical utility of LMS in the treatment of AA.

## Introduction

Aplastic anemia (AA) is well known as an immune-mediated bone marrow (BM) failure syndrome, characterized by hypocellularity and pancytopenia with fatty BM. Patients with AA often display multiple symptoms, including fatigue, purpura, or hemorrhage and increased susceptibility to infection, even leading to death (1, 2). AA can be causally associated with radiation, exposure to toxic drugs, or infection. Several studies indicated that abnormal immunity played an essential role in the pathogenesis of AA (3–5). Dysregulated T cells including the unbalanced polarization of T helper cell (Th)1/Th2, Th17/Regulator T cells (Tregs), and aberrant activated CD8^+^ cytotoxic T cells have been observed in most patients with AA (6). A variety of immune molecules such as IFN-γ, TNF-α, MIP-1α, and IL-2 (7) compose a cytokine network to destroy hematopoietic stem/progenitor cells (HSPC) and mesenchymal stem cells. As a result, immunity and inflammatory responses mediate the loss of HSPCs leading to an impaired BM microenvironment rather than “empty” BM (8, 9). To define the critical factors triggering AA, a murine model of AA was established by transfusing allogeneic lymph node (LN) cells into sub-lethally irradiated recipients, which elicited pancytopenia and death within 2–3 weeks. Importantly, disease progression and immunosuppressive treatment responses are similar to those observed in patients with AA (10).

Although the pathophysiology of AA has not been fully elucidated, it has been shown that up to 70% of the patients with AA would respond to immunosuppressive therapy (IST) such as anti-thymocyte globulin (ATG) and cyclosporin (CSA), which is a powerful evidence of immunopathogenesis. Furthermore, allogeneic hematopoietic stem cell transplantation is the preferred definitive treatment for AA (11). However, these treatments are not feasible for most patients with AA in developing countries due to the unavailability of HLA-matched donors or old age (>50 years old), high costs, and lack of health insurance services. In addition, the outcomes of AA if only treated with supportive care or CSA alone were not encouraging (12). Therefore, it is crucial to develop a novel regimen that had significant immunosuppressive effect, lower cost, and ease of administration for AA patients. Our previous study (13) reported that up to 66.4% of patients with severe AA achieved excellent responses at 12 months after using the novel immunosuppressive strategy of CSA combined with levamisole (CSA+LMS-based regimen). The incidence of overall survival and failure-free survival rates at 10 years were 60.2% and 48.3%, respectively, which has the preferable outcomes and lower cost compared to the ATG+CSA regimen. However, the molecular mechanisms involved remain unknown.

Levamisole (LMS), originally designed for anthelminthic applications, has subsequently been reported to have a wide range of immunomodulatory effects and is widely used in the adjuvant therapy of autoimmune diseases and malignant tumors (14, 15). Previous studies revealed that LMS promoted recovery of BM in patients who received chemotherapy by restoring the Th1/Th2 cell ratio to normal (16) and increasing the formation of granulocyte colony units (17). Efficacy of LMS treatment remains dose-dependent; which a low dose of LMS can induce dendritic cell maturation and stimulate T-cell activation but a high dose exerts inhibitory effects (18, 19). Moreover, LMS suppresses the adipogenesis of BM-derived mesenchymal stem cells from AA patients *via* the ZFP36L1–PPARGC1B axis (20).

Therefore, we postulated that the distinct complementary effects of LMS would result in further blockage of activated autoreactive T cells in AA. Here in this study, we found that LMS could improve hypocellularity and pancytopenia by regulating the function of T cells and antigen-presenting cells (APCs) in both patients with AA and immune-mediated murine BM failure models. Furthermore, the potential molecular mechanisms and the specific cellular targets in modulating the immune balance of LMS on AA treatment were identified.

## Materials and Methods

### Patient Enrollment and Data Collection

We retrospectively analyzed 28 patients with non-severe AA (NSAA) between 2006 and 2018 at the Institute of Hematology and Blood Diseases Hospital Chinese Academy of Medical Sciences and Peking Union Medical Colleges. All patients had a poor response with 3–6 months of standard CSA therapy but achieved a complete response (CR) or good partial response (GPR) after 3–6 months of the CSA+LMS-based regimen. We defined NSAA as hypocellular marrow with cytopenia in the peripheral blood (PB), which does not fulfill the criteria for severe AA [requiring at least two of the following: absolute neutrophil count (ANC) < 0.5×10^9^/L, platelet counts < 20×10^9^/L, or absolute reticulocyte count < 20×10^9^/L]. Patients with congenital AA, myelodysplastic syndrome (MDS), overt paroxysmal nocturnal hemoglobinuria, acute arrest of hemopoiesis, and significant comorbidities indicating imminent death were excluded. CR was defined as achieving an ANC > 1.5×10^9^/L, platelet count > 100×10^9^/L, and hemoglobin (Hb) > 100 g/L. GPR was defined by transfusion independence and an increase in hematological parameters from baseline values with at least Hb > 100 g/L, ANC > 1.0×10^9^/L, and platelet count > 50×10^9^/L. The CSA+LMS-based regimen was administered on alternate days for 12 more months (CSA 3–5 mg/kg per day, LMS 50 mg three times per day). Both CSA and LMS were followed by a slow tapering rate of 25% reduction in dose for every 3–4 months according to response, to reduce the risk of relapse. PB samples from patients with NSAA were collected *de novo de-novo*, 3–6 months of standard CSA therapy with poor response and CSA+LMS-based regimen with good response. Then, PB mononuclear cells (PBMCs) were separated by Ficoll-Hypaque density gradient centrifugation and were stained with the indicated antibodies on ice for 30 min by flow cytometry assay.

### Mice and Murine Model of AA

Eight-week-old C57BL/6 and CB6F1 mice were purchased from Beijing Huafukang lnc. and maintained in the specific pathogen-free barrier facilities. All experiments were performed in accordance with protocols approved by the Institutional Animal Care and Use Committee at the Institute of Hematology. As previously described (21), recipient CB6F1 mice were pre-irradiated with 5 Gy total body irradiation. Then, they were intravenously injected with 5×10^6^ LN cells from B6 donors after 4 to 6 h. Lymphocytes were isolated from inguinal, axillary, and lateral axillary LNs. The treated mice received LMS that dissolved in PBS at a daily dose of 20 mg/kg one time starting from day 0 (LN infusion) until the mice died. Mice who received vehicle treatment were used as controls. PB cell counts were detected on days 7, 10, and 14. Spleen cell subsets were detected by flow cytometry on day 14 after LN transfusion.

### Flow Cytometry and Cytokine Measurement

For the analysis of cell surface molecules, single-cell suspensions were prepared and fluorescently stained with the following antibodies from BioLegend: anti-human CD3 (317305), anti-human CD4(357146), anti-human CD8(344712), anti-human CD25(302609), anti-human CD56(362505), anti-mouse CD3(100203), anti-mouse CD4(100413), and anti-mouse CD8(100721). Tregs were first incubated with surface markers, washed, resuspended in 1 ml of cold Fixation/Permeabilization buffer (eBioscience), and incubated at 4°C for 1 h. After washing with 2 ml of permeabilization buffer (eBioscience), cells were stained with Foxp3 antibodies at 4°C for 50 min protected from light. Finally, cells were washed with 2 ml of permeabilization buffer and detected on FACS CantoII (BD Biosciences). All flow cytometer data were analyzed by the FlowJo software.

The concentrations of IL1/2/4/6/8/10 and TNF-α in the serum of patients were measured using the Bio-Plex Pro Human Cytokine 8-plex assay according to the manufacturer’s instructions.

### T-Cell Proliferation Assay

CD4^+^ CD25^-^ T cells and Tregs from the healthy donors were purified by the EasySep™ Human CD4^+^CD127^low^CD25^+^ Regulatory T Cell Isolation Kit (STEMCELL) and then labeled with cell trace™ violet dye, which is widely used to detect the cell proliferation according to the manufacturer’s protocol (Invitrogen). Anti-human CD3/28 antibodies (1μg/ml STEMCELL) and rhIL2 (100 ng/ml, PeproTech) were added into the cell culture medium. Different doses of LMS were added at the beginning of co-culture, and 72 h later, the proliferation of T cells was measured by flow cytometry.

### RNA Sequencing Assay

BM nucleated cells (BMNCs) from healthy donors and patients with NSAA at the time of initial diagnosis were separated, and then co-cultured with or without 40 μg/ml LMS for 48 h *in vitro*. Cells were harvested and total RNA was extracted. The RNA-sequencing assay was carried out using the Illumina HiSeq X platform, and the data obtained were analyzed by following the Hisat2 protocol (22).

### Virtual Docking Analysis

The establishment of a library of active binding sites for the reverse virtual screening based on well-characterized crystal structure proteins from PDB was previously reported (23, 24). The docking algorithm was described previously as well (25). In brief, the targeting protein library was established with a virtual screening module (http://www.vslead.com/index.php?r=vina/index) to perform the virtual screening. The targeting molecules were ranked according to the evaluated binding score.

### Cell Purification and Real-Time PCR (RT-PCR) Assay

Naïve CD4^+^T cells were isolated (purity > 95%) from wild-type C57BL/6 mice spleen using the Naive CD4^+^T Cell Isolation Kit (Miltenyi Biotec) according to the manufacturer’s instructions and were added with different doses of LMS or ruxolitinib (Rx) at the beginning of co-culture. Then, the cells were activated under the Th1 polarization condition [anti-CD3/28 antibodies (4 μg/ml), anti-IL-4 antibodies (10 μg/ml), and IL-12 (10 ng/ml) (BioLegend)] for 4 days. In some experiments, DC2.4 cells were stimulated by 1 μM TLR7 agonist (R848, from MCE) or DMSO (as a negative control) for 48 h. Total RNA after different treatments was extracted using Trizol as described by the manufacturer’s protocol, and retrotranscription was carried out using One-Step RT-PCR SuperMix (Transgen Inc.). The RT-PCR reaction system was sybergreen mixture from Transgene Inc. and the amplification step was 40 cycles including 94°C for 5 s, 55°C for 15 s, and 72°C for 10 s by the GeneAmp 7500 Sequence Detection System (Applied Biosystems). The RT-PCR primers used in this experiment are listed in **Supplementary Table 1**.

### Statistical Analysis

The Student’s *t*-test and the one-way ANOVA were performed using GraphPad Prism to calculate the statistical significance of the difference and *p*-values. Survival curves were evaluated using the Kaplan–Meier method and compared using a log-rank test. A *p*-value less than 0.05 was considered statistically significant. *p < 0.05; **p < 0.01; ***p < 0.001, ****p < 0.0001.

## Results

### Combination Treatment of CSA+LMS Was Effective in NSAA Patients With No Response to 3–6 Months of the Standard CSA Therapy

**Figure 1:**
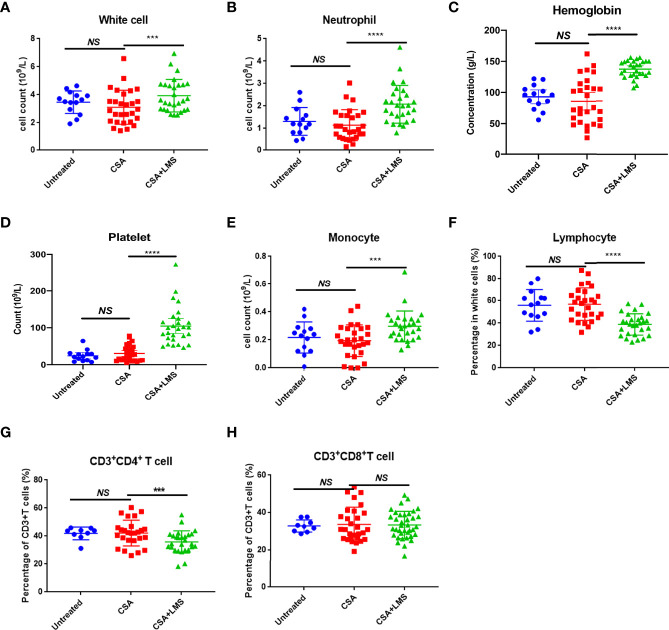
Difference in immune cell types in the peripheral blood of NSAA patients before and after the administration of the CSA+LMS-based regimen. Twenty-eight NSAA patients who achieved poor response after 3–6 months of standard CSA therapy but achieved complete response (CR) or good partial response (GPR) after 3–6 months of CSA+LMS-based regimen were enrolled. **(A–E)** The levels of white blood cells, neutrophils, hemoglobin, platelets, and monocytes in NSAA patients at *de novo* (untreated), the time point after 3–6 months of standard CSA therapy with poor response before administration of the CSA+LMS-based regimen (CSA treated) and the time point after the CSA+LMS-based regimen with good response (CSA+LMS). **(F–H)** The percentage of lymphocytes in whole white cells, CD3^+^CD4^+^ T cells in CD3^+^ T cells, and CD3^+^CD8^+^ T cells in CD3^+^ T cells in these three stages of the cohort. ***p < 0.001, ****p < 0.0001.

In this study, we collected 28 patients with NSAA who had no response after receiving 3–6 months of standard CSA therapy but achieved CR or GPR after 3–6 months of the CSA+LMS-based regimen. The counts/percentage of immune cells and the level of cytokines in the PB at *de novo*, the time point after 3–6 months of standard CSA therapy with poor response, and the time point after the CSA+LMS-based regimen with good response were statistically analyzed, including white blood cells, neutrophils, Hb, platelets, reticulocytes, monocytes, lymphocytes, CD19^+^ cells, CD3^+^ cells, CD3^+^CD4^+^ cells, CD3^+^CD8^+^ cells, CD3^+^CD56^+^ cells, CD3^-^CD56^+^ cells, Tregs, IL1, IL2, IL4, IL6, IL8, IL10, and TNF-α. We compared the indicators mentioned above at the time of initial diagnosis and after the standard CSA treatment for 3–6 months. White blood cells, neutrophils, Hb, platelets, and reticulocytes showed no improvement after the single use of CSA, as well as the other indicators in these patients (**Figure 1**). However, we found that the counts/percentages of white blood cells (**Figure 1A**), neutrophils (**Figure 1B**), Hb (**Figure 1C**), platelets (**Figure 1D**), and reticulocytes (**Supplementary Figure 1A**) were significantly elevated after CSA+LMS treatment, which demonstrated the efficacy of CSA+LMS treatment on NSAA patients. As summarized previously, in the PB from acquired AA patients, DCs, CD4^+^Th1 cells, and CD8^+^ T cells were observed increased, while granulocytes, monocytes, NKs, B cells, and Tregs decreased (1). In our study, the CSA+LMS treatment could elevate the monocyte count (**Figure 1E**) and reduce the percentages and absolute numbers of lymphocytes (**Figure 1F**, **Supplementary Figure 1C**), especially CD4^+^ T cells (**Figure 1G**), but not influence NK cells, NK/T cells, B cells, CD8^+^ T cells, and Treg percentages (**Figure 1H**, **Supplementary Figures 1D–H**). By comparing cytokine levels before and after CSA+LMS treatment, no significant difference was observed on IL1, IL2, IL4, IL6, IL8, IL10, and TNF-α (**Supplementary Figure1I**).

### LMS Monotherapy Improved Hemocytopenia and Prolonged the Survival in the Immune-Mediated BM Failure Murine Model

**Figure 2:**
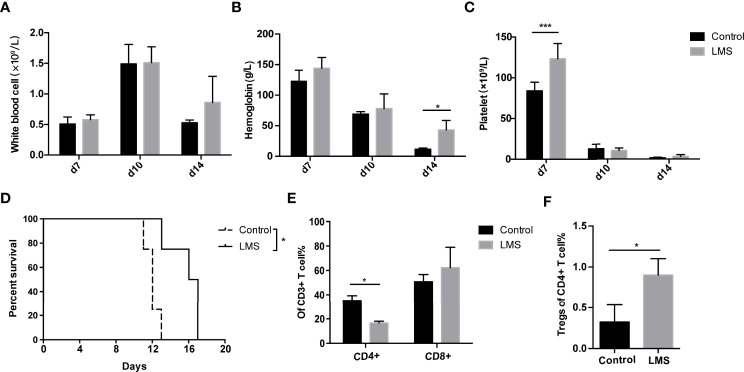
LMS improved pancytopenia and prolonged animal survival in an immune-mediated bone marrow failure murine model. Irradiated CB6F1 recipients were transplanted with C57BL/6 lymph node cells with PBS or 20 mg/kg LMS oral daily administration. **(A–C)** The white blood cell count, hemoglobin, and platelet levels in the peripheral blood from control and LMS-treated groups were measured at days 7, 10, and 14 (Control group: *n* = 4; LMS-treated group: *n* = 4). **(D)** Overall survival was shown in each group (Control group: *n* = 5; LMS-treated group: *n* = 5). **(E)** The percentage of CD4^+^ and CD8^+^ T cells in CD3^+^ T cells in the spleens of two groups on day 14 (*n* = 3). **(F)** The percentages of CD4^+^Foxp3^+^ Tregs in the spleens of two groups on day 14 (*n* = 3). *p < 0.05; **p < 0.01; ***p < 0.001.

To confirm the effects of LMS on AA, we established an immune-mediated BM failure murine model as described previously (26). Compared to the control group, the LMS treatment group had partially increased platelet count on day 7 and Hb level on day 14 after LN cell infusion (**Figures 2B, C**). However, the white blood cells in the LMS treatment group were barely altered compared to the control group (**Figure 2A**). In the parallel survival experiment, vehicle-treated mice with BM failure died within 2 weeks after the LN cell infusion, while LMS treatment could effectively prolong the survival of mice (**Figure 2D**). Furthermore, the spleens from control and LMS-treated groups were separated on day 14 and the subsets of T cells were detected by flow cytometry. As shown in **Figure 2E**, LMS treatment could effectively reduce the percentage of CD4^+^ T cells rather than CD8^+^ T cells. Furthermore, the percentage of CD4^+^Foxp3^+^ Tregs was significantly increased in the spleen of the LMS treatment group than in the control group (**Figure 2F**).

### LMS Inhibited the Proliferation of CD4^+^ Responder T Cells and Elevated the Proportion of Tregs After Stimulation by Anti-CD3/28 Antibody *In Vitro*

**Figure 3:**
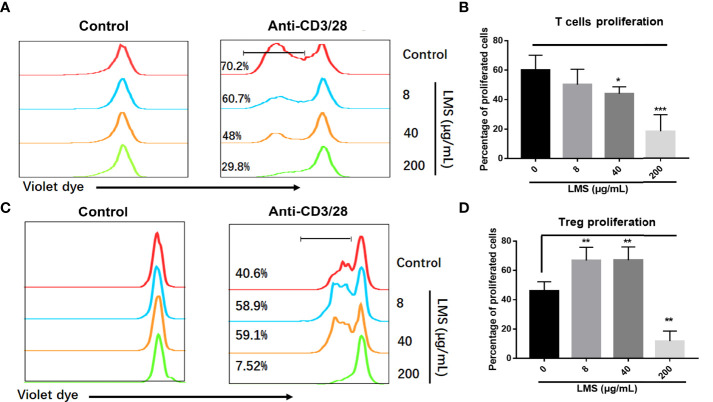
LMS suppressed the proliferation of T cells and elevated the proportion of Tregs under anti-CD3/38 antibody stimulation *in vitro*. Human CD4^+^ CD25^-^ T cells and Tregs purified from peripheral blood mononuclear cells of healthy donors were stained with cell trace violet dye to track cell proliferation. Various concentrations of LMS (0, 8, 40, and 200 μg/ml) and anti-CD3/28 antibody (1μg/ml) were added into the culture medium. The proliferation of violet dye-labeled responder T cells **(A, B)** and Tregs **(C, D)** were detected and statistically analyzed by flow cytometry after 72 h of stimulation. *p < 0.05; **p < 0.01; ***p < 0.001. All data represent at least two to three independent experiments.

We have demonstrated the efficacy of immune regulation of LMS on AA through retrospective analysis and a murine model of immune-mediated BM failure. To investigate the role of LMS on T cells, we further verified whether LMS could directly influence T cells. CD4^+^CD25^-^ T cells and Tregs were purified from healthy donors and labeled with cell trace violet dye. T-cell subsets were stimulated with 1μg/ml anti-CD3/28 antibodies for 72 h with or without different concentrations of LMS (8 μg/ml, 40 μg/ml, and 200 μg/ml), and the proliferation was detected by flow cytometry. As shown in **Figures 3A, B**, the proliferation of activated T cells was significantly suppressed by LMS treatment, while the percentage of Tregs was elevated with low and medium doses of LMS treatment (**Figures 3C, D**). These results suggested that LMS had a direct effect on T cells in achieving immunoregulatory activity.

### LMS Mainly Downregulated Molecules Related to T Cells and APCs in NSAA Patients by Transcriptional Analysis

**Figure 4:**
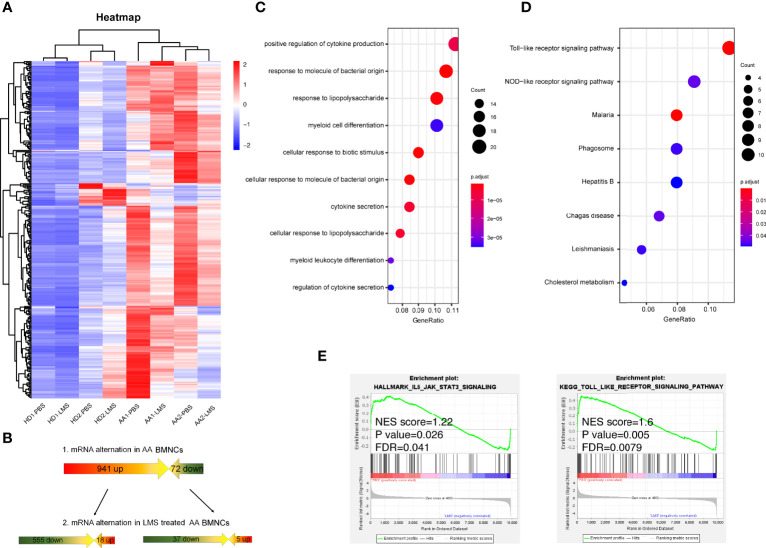
RNA sequencing indicating LMS-induced changes in BMNCs from NSAA patients could alter multiple immune-related functions and signaling pathways. **(A, B)** BMNCs from healthy donors or NSAA patients were separated and treated with PBS or 40 μg/ml LMS. After co-culturing *in vitro* for 48 h, total RNA was extracted and the RNA sequencing was carried out. Gene transcripts with higher expression in NSAA and lower levels after LMS treatment were selected and analyzed. Dot graph showed GO pathways **(C)** and KEGG pathways **(D)** downregulated in patients with NSAA treated with LMS compared with those treated with PBS *in vitro*. **(E)** Gene pathways that were differentially expressed in NSAA patients with or without LMS according to GSEA (right side was post-treated sample by LMS). Gene sets were considered statistically significant at NES<1, FDR<0.05 and p value <0.05.

To explore the possible mechanisms of LMS-induced effects on the BM immune cells in AA patients, we analyzed the transcriptome of BMNCs incubated with or without 40 μg/ml LMS for 48 h *in vitro* sorted from healthy donors and NSAA patients. To determine the impact of LMS treatment, we identified genes whose transcriptional expression was significantly upregulated in NSAA samples and inhibited by LMS treatment, as well as genes whose expression was significantly downregulated in NSAA samples and upregulated by LMS treatment compared with those of healthy donors (**Figures 4A, B**). As summarized in **Table 1**, genes whose transcriptional expression was significantly inhibited by LMS in NSAA samples were mainly related to APCs and T cells and partially related to B cells, NK cells, and neutrophils. Regarding function, genes related to metabolism and migration were mainly affected by LMS treatment.

**Table 1:** The effects of LMS on immune cells in patients with AA.

APCs	Neutrophil	B cell	T cell	NKcell	metabolism	migration	Epi-genetic	Transport/ATP/energy	platelet	Cell cycle/proliferation/Survival /apoptosis
CSF1R FUCA1 HLA-DQB1 CXCL16 ADA2 P2RY8 GLIRP1 PSMB10 CX3CR1 SAMHD1 PILRA ENG TMEM176B MPEG1 IL10RA FCGR3A CD14 IL32 DEFA1B	FUCA1 DEFA1B	HLA-DQB1 FAM117A BCL2L1 DEFA1B	LY6E CDSW FAM117A IL32 BCL2L1 GNLY DEFA1B	GZMH CTSW IL32 GNLY DEFA1B	IDH2 CRIP1 GSR APOBEC3G GLIPR1 APRT PSMB10 HK1 LIPA OAZ2 8-Mar CASP8 SELENBP1 UCP2 BCL2L1 TMCC2 TXNIP	THBS1 CTNNA1 ADA2 LY6E SH3BP1 AHNAK DMTN ITGAL	EID1 HIST1H1C RARRES3 STAT1	CYTH4 OSBP1 TC2N DCAF12 NCOA4	PLA2G7	
CCL4L2 CCRL2 CD83 CCL3 CCL4 CD48 IL1B CCL24 SLAMF7 CCL2	CCRL2 PI3 CXCL8	CD48	CCL4 CCL24 SLAMF7 CD48	SLAMF7	USP12 ACSL5 PLIN2 AKR1B1 GM2A SERPINB2 CTSL SPINK1 DBI		MTERF4 ZBTB1 BHLHE40 XBP1 CIR1	XPOT ATP13A3 NDUFS6 FRG1 ATP13B3 ARF4 TFRC AQP9 CSTB LGALS3	C15orf48	PIM3 MSL2 EMP1 CCNG1 DAD1 PP1R15A ANXA5 IL1B

Compared with the expression of genes from healthy donors, the significant upregulated genes in NSAA samples and inhibited by LMS treatment were summarized in the green-filled box. In addition, the significant downregulated genes in NSAA samples while upregulated by LMS treatment were summarized in the orange-filled box.

Pathway enrichment analysis was performed by the Gene Oncology (GO), Kyoto Encyclopedia of Genes and Genomes (KEGG) pathway database, and gene set enrichment analysis (GSEA). As a result, the regulation of cytokine secretion, myeloid differentiation, Toll like receptors (TLR), and NOD-like receptor signaling pathways in patients with NSAA were suppressed by LMS treatment *in vitro* (**Figures 4C, D**). Pathways involved in IL-6/STAT3 and TLR were significantly downregulated in BMNCs treated with LMS in patients with NSAA *via* GSEA (**Figure 4E**). These results indicated that the effect of LMS on immune cells in BM from NSAA patients might be responsible for the therapeutic efficacy of LMS in NSAA.

### JAK1/2 and TLR7 Were the Targeting Molecules of LMS Predicted by the Virtual Docking Algorithm

**Figure 5:**
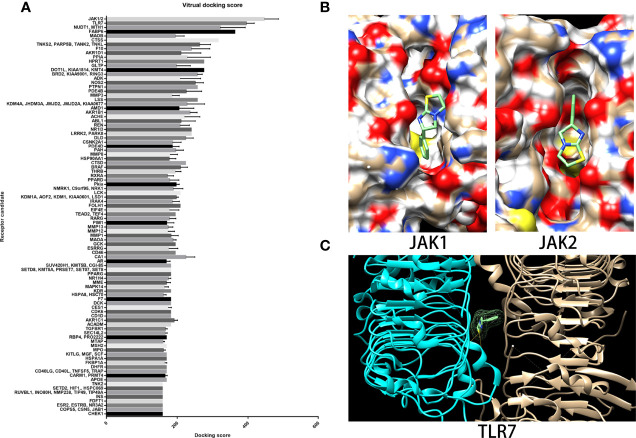
JAK1/2 and TLR7 were predicted binding targets with relatively high binding scores *via* virtual docking assay. **(A)** The target library and docking algorithm developed by the Vslead team were employed in this study, and the target library was used to screen the potential binding targets of LMS, which ranked them according to the binding score. **(B)** The binding site and position of LMS on the crystal structure of JAK1 (5e1e) and JAK2 (3eyg) were analyzed *via* Dock6 and presented: two H-bonds were indicated. **(C)** The crystal structure of the TLR7 complex (6if5) was shown and the binding site of LMS to TLR7 was analyzed by Dock6.

As a drug that is well-known as an antiparasitic agent, the novel effects of LMS on NSAA treatment may rely on a new unidentified molecular target. In the search for potential targets, we employed the virtual docking assay, which was established based on the PBD protein crystal structure library (https://www.rcsb.org/) and the virtual docking algorithm (Matching algorithms, MA) through vina and dock6. As summarized in **Figure 5A**, Janus kinase1/2 (JAK1/2) had the highest docking score to LMS followed by the TLR7 complex. Compared to the positive antagonists of these candidate targets, we found that LMS could competitively bind to the specific antagonist binding site in JAK1/2 containing two H-bonds (**Figure 5B**). The JAK/signal transducers and activators of transcription (JAK/STAT) signaling pathway is strongly associated with the immune response and regulation of numerous cytokines secretion, which might dominate the activity of LMS function on the BMNCs in patients with NSAA. Moreover, given that the TLR signaling pathway could be altered by exposure to LMS as mentioned above, we attempted to dock the active binding sites of TLR family members with LMS and found that LMS could dock to the TLR7 complex with a higher docking score and involved two H-bonds (**Figure 5C**).

### Downstream of the JAK/STAT and TLR Signaling Pathways Could Be Directly Inhibited by LMS *In Vitro*

**Figure 6:**
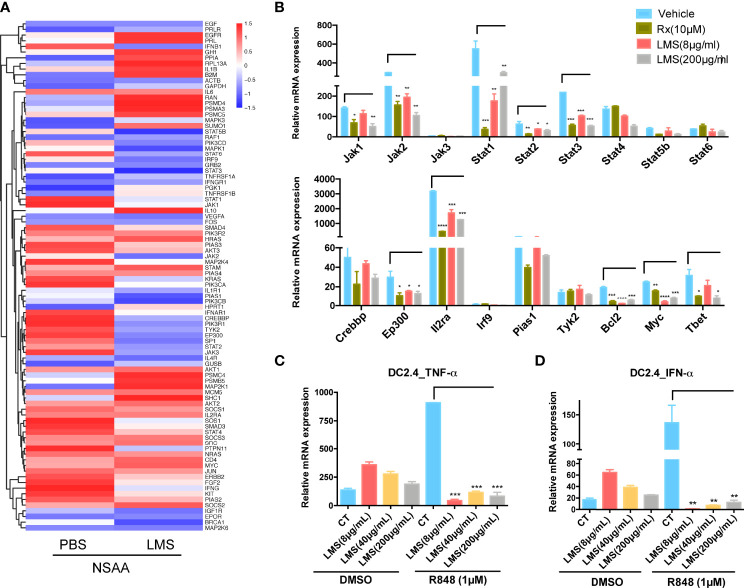
LMS downregulated key molecules related to TLR and JAK/STAT signaling pathways directly *in vitro*. **(A)** Heatmaps showed the TLR and JAK/STAT signaling pathway-related genes differentially expressing based on their function in NSAA patients treated with PBS or 40 μg/ml LMS for 48 h *in vitro* according to RNA sequencing (PBS: *n* = 2; LMS: *n* = 2); **(B)** *In vitro* purified naive CD4^+^ T cells from wild-type C57BL/6 mice were treated with or without different doses of LMS (8 μg/ml, 200 μg/ml) or 10 μM ruxolitinib (Rx) under the Th1 polarization conditions [anti-CD3/28 antibodies (4 μg/ml), anti-IL-4 antibodies (10 μg/ml), and IL-12 (10 ng/ml)] for 4 days. The mRNA expression of molecules related to the JAK/STAT signaling pathway in these cells was detected by RT-PCR (*n* = 3). **(C, D)** The mRNA expression of IFN-α and TNF-α was shown in DC2.4 cells with or without different doses of LMS (8 μg/ml, 40 μg/ml, and 200 μg/ml) under 1 μM R848 stimulation *in vitro* for 48 h by RT-PCR (*n* = 3). *p < 0.05; **p < 0.01; ***p < 0.001, ****p < 0.0001. All data represent at least three independent experiments.

To investigate the underlying molecular mechanisms by which LMS induces immunoregulation, we further inspected the involved genes related to the JAK/STAT and TLR signaling pathways based on the virtual docking analysis. As shown in **Figure 6A**, the expression of JAK/STAT-associated genes (e.g., *JAK1/2/3, TYK2, STAT1/3/5/6, CREBBP, EP300, PIAS*, and *SOS*) and TLR-associated genes (e.g., *JUN, MAPK, PIK3CA/B, AKT3, IFNAR1*, and *IFNB1*) were inhibited in BMNCs of two patients with NSAA following LMS treatment *in vitro* by transcriptional analysis. To verify the findings, naive CD4^+^ T cells from wild-type C57BL/6 mice were stimulated with anti-CD3/CD28 in the presence or absence of indicated concentrations of LMS or ruxolitinib (Rx) which is a JAK1/2 inhibitor used as a positive control under the Th1 polarizing condition (IL12 and anti-IL4). The mRNA expression levels of *Jak1/2, Stat1/3, Ep300, Bcl-2, Myc*, and *T-bet* related to the JAK/STAT signaling pathway were decreased *in vitro* in LMS-treated CD4^+^ T cells, which showed comparable results to Rx-treated CD4^+^ T cells (**Figure 6B**). These results indicated that LMS may partly impact the functional activity of CD4^+^ T cells by inhibiting the activation of the JAK/STAT signaling pathway. In addition, we used TLR7 agonists (R848) to stimulate the murine dendritic cell line, DC2.4, with or without different doses of LMS pretreatment. We then detected the downstream production of proinflammatory cytokines related to the TLR signaling pathway. As shown in **Figures 6C, D**, the production of IFN-α and TNF-α under the stimulation of R848 was obviously inhibited by LMS treatment.

## Discussion

In this study, we demonstrated that the CSA+LMS-based regimen might be a promising strategy for AA patients who received 3–6 months of standard CSA therapy with poor response. Additionally, LMS effectively improved the survival of the immune-mediated murine BM failure model and showed high efficacy in suppressing CD4^+^ T cells and stimulating immunosuppressive Tregs *in vivo* and *in vitro*. Our data indicate that regulation of JAK/STAT and TLR7 activity and its downstream signaling molecules are pivotal to the therapeutic effect of LMS.

LMS was primarily developed as an anthelminthic agent and a cocaine adulterant. The effect of LMS on colony-forming T lymphocyte cells was determined by the number or function of plastic-adherent mononuclear cells *in vitro* (27). With regard to the effect of LMS on AA *in vivo*, we previously found that the combination of low dose of CSA and LMS achieved a similar CR response to the CSA monotherapy (28). Moreover, we previously explored the effect of the CSA+LMS regimen on moderate AA (29), refractory or relapsed severe AA (30), and SAA (31) and confirmed the efficacy of LMS in AA treatment. The CSA+LMS-based regimen lowered the treatment cost and avoided the side effects caused by long-term high-dose application of CSA on the premise of therapeutic efficacy. In this study, we found that the CSA+LMS-based regimen could improve response rates for AA patients who received 3–6 months of standard CSA therapy with poor response, suggesting that LMS may have different immunoregulatory effects from CSA in the pathogenesis of AA. However, the direct molecular targets and the immune cell subsets affected by LMS are still unknown. T cells have been confirmed to play an important role in the pathogenesis of acquired AA and are significantly increased in the residual hematopoietic area from BM of AA patients (32). Furthermore, Treg defection may diminish the immune regulation of T lymphocytes and both the frequency and absolute number of Tregs were reduced in newly diagnosed patients with AA (33) but recovered after IST treatment (34). In our study, LMS improved pancytopenia and BM failure by suppressing effector T cells and expanding Tregs *in vivo*. The results are consistent with our *in vitro* experiment showing that LMS could suppress CD4^+^ T cell proliferation and promote Treg expansion directly under the stimulation of the T-cell receptor. However, LMS administration alone provided moderate benefit and prolonged survival in just a few days in the murine model. We hypothesized that the immunoregulatory effects of LMS were not sufficient to prevent the progression of AA, but the combination of LMS with other immunosuppressive treatment may supplement the pharmacological effects of this therapy. Furthermore, the pathogenesis of AA is not caused by abnormal activity of a single signaling pathway. LMS can act on a variety of immune cells, and similar regulatory effects on multiple molecules in the same pathway are the foundation of its activity, which also weakened its efficacy as a single drug in the treatment of AA to some extent.

To explore the underlying molecular mechanisms and potential cellular targets of LMS, we focused on the TLR and JAK/STAT signaling pathways by the virtual docking assay and transcriptional analysis. The TLR signaling pathway participates in the innate immune response and may induce the pathogenesis of AA by triggering adaptive immunity (35, 36). Young et al. (37) found that TLRs presented increased mRNA expression in the CD4^+^ T cells and also had a similar response on the CD8^+^ T cells from AA patients. TLR activation triggers the release of cytokines and may induce the adaptive immune response of the Th1 cells (38,39). In this study, LMS could reduce the IFN-α and TNF-α mRNA expression related to the TLR signaling pathway of DC2.4 cells by activating TLR7. These data indicated that the innate immune system plays a major role in the pathophysiology of AA.

The JAK/STAT signaling pathway has also been reported to be involved in the regulation of CD4^+^ T cells and cytokine secretion, which are critical participants in inflammatory responses (40). Stat3, which is crucial for IL6 and IL23 signaling during Th17 differentiation (41), has been shown to be negatively regulated by LMS in patients with AA in our study. JAK1/2-related IL12 and IFN-γ signaling pathways subsequent to Stat1 and Stat3 activation are essential for polarization of Th1 cells (42). In addition, in this study, LMS significantly inhibited the expression of *Jak1/2, Stat1/3*, and its downstream *Ep300, Bcl-2*, and *Myc* mRNA expression in CD4^+^ T cells under activating conditions. Furthermore, LMS could downregulate the key transcription factors of Th1 cells (T-bet), which produced a beneficial effect on controlling Th1 differentiation. These effects may partially explain the molecular mechanism triggered by LMS in the treatment of AA. To provide stronger evidence of LMS targeting TLRs and JAKs, more functional analysis and molecular biochemistry experiments should be performed.

Given the multiple mechanisms underlying the AA including metabolism disorder and irregular immune activation, drugs targeting the single molecule seem insufficient to reverse the progression of AA. In this study, we found that LMS could suppress CD4^+^ T cells and stimulate immunosuppressive Tregs both in the retrospective clinical study based on patients with AA and in a murine model of BM failure. JAK1/2 and TLR7 are the potential binding targets of LMS in modulating the immune balance by virtual docking, and these targets were further verified by specific pharmacological antagonist assays in target cells. Therefore, the chemical optimization of LMS based on the targeting molecules in this study provides a potential therapeutic strategy for AA.

## Data Availability Statement

The data presented in the study are deposited in the European Nucleotide Archive (ENA) repository, accession number PRJEB54093.

## Ethics Statement

The studies involving human participants were reviewed and approved by Ethics Committee of the Institute of Hematology, Chinese Academy of Medical Sciences (Ethical approval No.HG2020014-EC-1). The patients/participants provided their written informed consent to participate in this study. The animal study was reviewed and approved by the Institutional Animal Care and Use Committee at the Institute of Hematology, Chinese Academy of Medical Sciences.

## Author Contributions

XP and JW performed experiments, analyzed the data, and wrote the manuscript. JL, MW, FZ, and LL performed partial experiments. MG, EJ, SF, and YZ helped collect the samples and analyzed the data. MH, XP, and YZ designed the study and directed the experiments. All authors contributed to the article and approved the submitted version.

## Funding

This work was supported by the National Natural Science Foundation of China (81700099) and the PUMC Graduate Innovation Fund (2019-1002-84).

## Conflict of Interest

The authors declare that the research was conducted in the absence of any commercial or financial relationships that could be construed as a potential conflict of interest.

## Publisher’s Note

All claims expressed in this article are solely those of the authors and do not necessarily represent those of their affiliated organizations, or those of the publisher, the editors and the reviewers. Any product that may be evaluated in this article, or claim that may be made by its manufacturer, is not guaranteed or endorsed by the publisher.

## Supplementary Material

Supplementary Figure 1 Peripheral blood cell changes and cytokine secretion in serum in NSAA patients before and after receiving CSA+LMS based regimen. **(A–C)** Reticulocytes counts, the percentage of monocytes and lymphocyte counts in NSAA patients at *de-novo* (untreated), the time point after 3-6 months of standard CSA therapy with poor response before administration of CSA+LMS based regimen (CSA treated), and the time point after the CSA+LMS based regimen with good response (CSA+LMS). **(D–H)** Percentages of B cells, NK cells, NK/T cells, CD3^+^ T cells and Tregs in these three stages in this cohort by flow cytometric analysis. **(I)** Cytokine secretion in the serum in the CSA treated and CSA+LMS stages in this cohort. *P< 0.05, ****P<0.0001.
