# Supplementary figures and images for: Levamisole Suppresses CD4+ T-Cell Proliferation and Antigen-Presenting Cell Activation in Aplastic Anemia by Regulating the JAK/STAT and TLR Signaling Pathways
Source: Front Immunol. 2022 Jul 14;13:907808. doi: 10.3389/fimmu.2022.907808 (PMC9331934; doi:10.3389/fimmu.2022.907808)

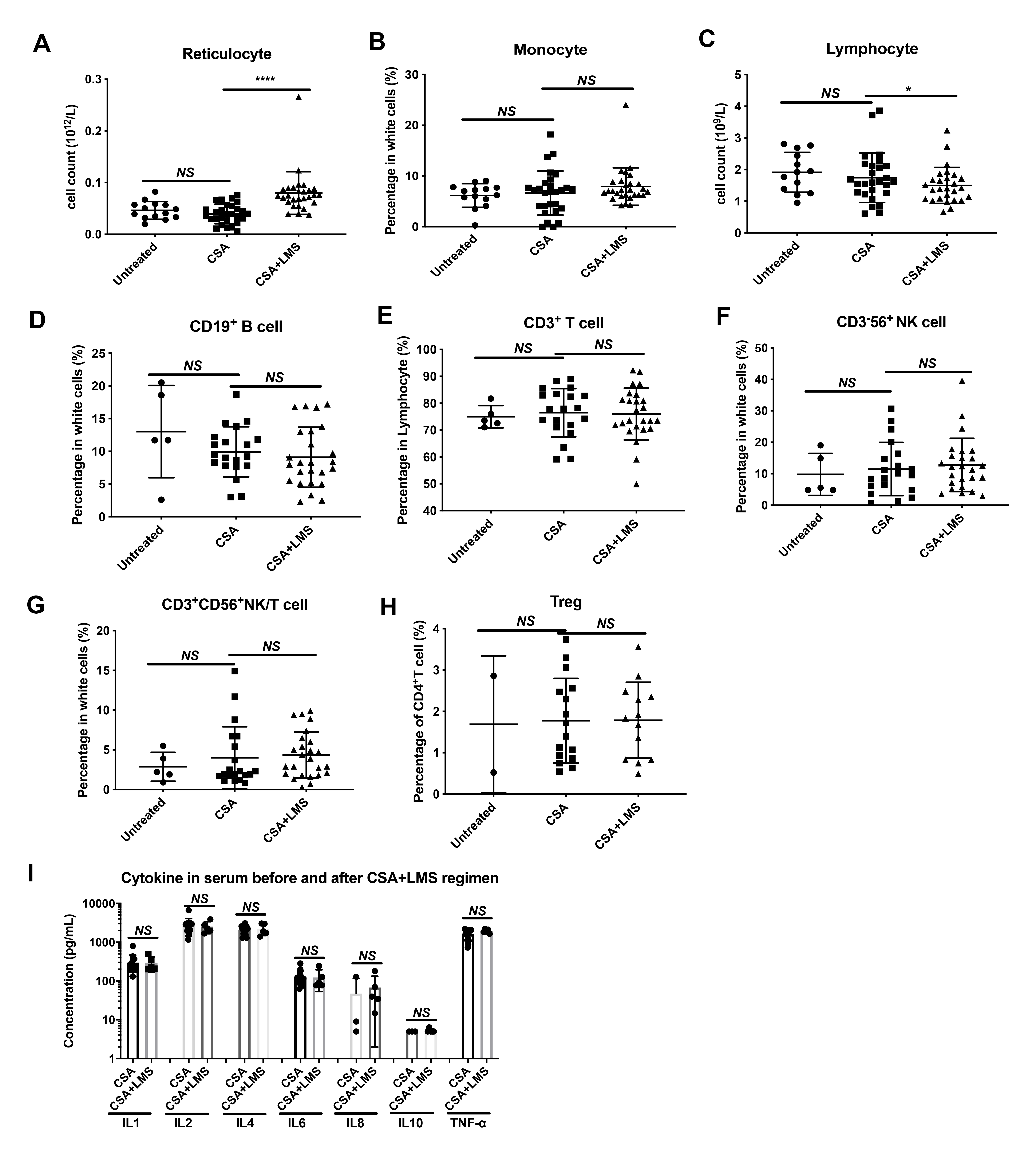

Supplement: Supplementary Figure 1 — Peripheral blood cell changes and cytokine secretion in serum in NSAA patients before and after receiving CSA+LMS based regimen. (A–C) Reticulocytes counts, the percentage of monocytes and lymphocyte counts in NSAA patients at de-novo (untreated), the time point after 3-6 months of standard CSA therapy with poor response before administration of CSA+LMS based regimen (CSA treated), and the time point after the CSA+LMS based regimen with good response (CSA+LMS). (D–H) Percentages of B cells, NK cells, NK/T cells, CD3+ T cells and Tregs in these three stages in this cohort by flow cytometric analysis. (I) Cytokine secretion in the serum in the CSA treated and CSA+LMS stages in this cohort. *P< 0.05, ****P<0.0001. [file Image_1.tif]
