# Supplementary material for: Levamisole Suppresses CD4+ T-Cell Proliferation and Antigen-Presenting Cell Activation in Aplastic Anemia by Regulating the JAK/STAT and TLR Signaling Pathways
Source: Front Immunol. 2022 Jul 14;13:907808. doi: 10.3389/fimmu.2022.907808 (PMC9331934; doi:10.3389/fimmu.2022.907808)
Supplement: Supplementary file 2 [file Table_1.docx]

**Table S1 Primer sequences for RT-PCR**

| Primer Name | Sequence (5’-3’) |
| --- | --- |
| mouse-Jak1_F | CTCTCTGTCACAACCTCTTCGC |
| mouse-Jak1_R | TTGGTAAAGTAGAACCTCATGCG |
| mouse-Jak2_F | TTGTGGTATTACGCCTGTGTATC |
| mouse-Jak2_R | ATGCCTGGTTGACTCGTCTAT |
| mouse-Jak3_F | GGCGTGGCGGTTAGTAAAGAA |
| mouse-Jak3_R | CCCCCTATCTAGTCTCACCCT |
| mouse-Stat1_F | TCACAGTGGTTCGAGCTTCAG |
| mouse-Stat1_R | GCAAACGAGACATCATAGGCA |
| mouse-Stat2_F | TCCTGCCAATGGACGTTCG |
| mouse-Stat2_R | GTCCCACTGGTTCAGTTGGT |
| mouse-Stat3_F | AGCTGGACACACGCTACCT |
| mouse-Stat3_R | AGGAATCGGCTATATTGCTGGT |
| mouse-Stat4_F | TGGCAACAATTCTGCTTCAAAAC |
| mouse-Stat4_R | GAGGTCCCTGGATAGGCATGT |
| mouse-Stat5_F | CGATGCCCTTCACCAGATG |
| mouse-Stat5_R | AGCTGGGTGGCCTTAATGTTC |
| mouse-Stat6_F | CTCTGTGGGGCCTAATTTCCA |
| mouse-Stat6_R | CATCTGAACCGACCAGGAACT |
| mouse-Crebbp_F | GGCTTCTCCGCGAATGACAA |
| mouse-Crebbp_R | GTTTGGACGCAGCATCTGGA |
| mouse-Ep300_F | TTCAGCCAAGCGGCCTAAA |
| mouse-Ep300_R | CGCCACCATTGGTTAGTCCC |
| mouse-IL2ra_F | AACCATAGTACCCAGTTGTCGG |
| mouse-IL2ra_R | TCCTAAGCAACGCATATAGACCA |
| mouse-Irf9_F | CAGCAGGAACCCTCCCTAAC |
| mouse-Irf9_R | GAAAGGCCACACACCTGAGTT |
| mouse-Pias1_F | GCGGACAGTGCGGAACTAAA |
| mouse-Pias1_R | ATGCAGGGCTTTTGTAAGAAGT |
| mouse-Tyk2_F | AGCCATCTTGGAAGACAGCAA |
| mouse-Tyk2_R | GACTTTGTGTGCGATGTGGAT |
| mouse-Bcl2_F | GTCGCTACCGTCGTGACTTC |
| mouse-Bcl2_R | CAGACATGCACCTACCCAGC |
| mouse-Tbet-F | AGCAAGGACGGCGAATGTT |
| mouse-Tbet-R | GGGTGGACATATAAGCGGTTC |
| mouse-Myc_F | ATGCCCCTCAACGTGAACTTC |
| mouse-Myc_R | CGCAACATAGGATGGAGAGCA |
| mouse-Ifn-α_F | TGTGACCTTCCTCAGACTC |
| mouse-Ifn-α_R | TCATTTGTACCAGGAGTGTC |
| mouse-Tnf-α_F | TCCCTCTCATCAGTTCTA |
| mouse-Tnf-α_R | TTGAGATCCATGCCGTTG |
| mouse-β-actin_F | GGCTGTATTCCCCTCCATCG |
| mouse-β-actin_R | CCAGTTGGTAACAATGCCATGT |
